# Thiol-Activated Hydrogen Sulfide Donors Antiviral and Anti-Inflammatory Activity in Respiratory Syncytial Virus Infection

**DOI:** 10.3390/v10050249

**Published:** 2018-05-10

**Authors:** Nikolay Bazhanov, Teodora Ivanciuc, Haotian Wu, Matteo Garofalo, Jianming Kang, Ming Xian, Antonella Casola

**Affiliations:** 1 Department of Pediatrics, University of Texas Medical Branch at Galveston, Galveston, TX 77555, USA; nibazhan@utmb.edu (N.B.); teivanci@utmb.edu (T.I.); hwof08@gmail.com (H.W.); mpgarofa@utmb.edu (M.G.); 2 Department of Chemistry, Washington State University, Pullman, WA 99164, USA; jianming.kang@wsu.edu (J.K.); mxian@wsu.edu (M.X.); 3 Department of Microbiology and Immunology, University of Texas Medical Branch at Galveston, Galveston, TX 77555, USA

**Keywords:** respiratory syncytial virus, hydrogen sulfide donor, airway inflammation, mouse model

## Abstract

We have recently shown that endogenous hydrogen sulfide (H_2_S), an important cellular gaseous mediator, exerts an antiviral and anti-inflammatory activity in vitro and in vivo, and that exogenous H_2_S delivered via the synthetic H_2_S-releasing compound GYY4137 also has similar properties. In this study, we sought to extend our findings to a novel class of H_2_S donors, thiol-activated gem-dithiol-based (TAGDDs). In an in vitro model of human respiratory syncytial virus (RSV) infection, TAGDD-1 treatment significantly reduced viral replication, even when added up to six hours after infection. Using a mouse model of RSV infection, intranasal delivery of TAGDD-1 to infected mice significantly reduced viral replication and lung inflammation, markedly improving clinical disease parameters and pulmonary dysfunction, compared to vehicle treated controls. Overall our results indicate that this novel synthetic class of H_2_S-releasing compounds exerts antiviral and anti-inflammatory activity in the context of RSV infection and represents a potential novel pharmacological approach to ameliorate viral-induced lung disease.

## 1. Introduction

Hydrogen sulfide (H_2_S) is an endogenous gaseous transmitter that acts as an intracellular messenger molecule, playing physiological roles in a variety of functions such as synaptic transmission, vascular tone, angiogenesis, inflammation, and cellular signaling. In the respiratory system, changes in H_2_S cellular levels have been associated with the pathophysiology of acute and chronic lung diseases such asthma, chronic obstructive pulmonary disease, pulmonary fibrosis and hypoxia-induced pulmonary hypertension, as it regulates airway bronchoconstriction, pulmonary circulation, fibrosis, oxidative stress, and inflammation (reviewed in [1]). In mammals H_2_S is produced primarily by three enzymes: cystathionine-γ-lyase (CSE), cystathionine-β-synthase (CBS), and 3-mercaptopyruvate sulfurtransferase (MST) [2,3,4]. H_2_S donors have been used to demonstrate how therapeutic H_2_S administration exert significant effects in various animal models of inflammation, reperfusion injury and circulatory shock [5]. As a number of airway diseases are associated with lower H_2_S cellular levels, the use of H_2_S donors may represent an important therapeutic approach to increase H_2_S tissue levels. A number of H_2_S donors exist that include inorganic salts, compounds with organic backbones, amino-acids, and naturally occurring compounds. Sulfide salts, such as sodium hydrosulfide (NaHS) and sodium sulfide (Na_2_S), represent the only donor class that has been extensively used in a variety of disease models by testing their efficacy in cells, tissues, and animals. These salts generate a large increase of H_2_S in a short time period, leading to potential toxic effects. On the other hand, naturally occurring and lab-produced H_2_S donors such as garlic extracts or derivatives of phosphorodithioate thioaminoacids exhibit a slow and more controlled H_2_S release that mimic more physiological settings (reviewed in [6,7]).

In recent studies we found that endogenous H_2_S has an antiviral and anti-inflammatory role against respiratory syncytial virus (RSV) [8,9], which represent a major cause of pediatric upper and lower respiratory tract infections [10]. They are associated with bronchiolitis, pneumonia and flu-like syndromes, as well as asthma exacerbations, and represent a substantial public health problem for the community. Using an in vitro model of airway epithelial cell infection, we found that administration of H_2_S donor GYY4137, a water-soluble H_2_S donor that shows a slow (in the micromolar range when used at millimolar concentrations) release of H_2_S by hydrolysis in a pH-dependent manner [11], strongly inhibited replication of RSV, as well as other RNA enveloped viruses [8,12]. In a mouse model of RSV infection, administration of GYY4137 resulted in a significant reduction of lung viral titers and airway inflammation, and in an improvement of lung function and disease outcome [9]. Although a number of H_2_S donors have been reported, donors with controllable H_2_S release capability are still very limited. To broaden our initial observations, we sought to assess the antiviral effects of H_2_S using a different family of donors—Thiol-Activated *gem*-Dithiol-based H_2_S donors (TAGDD) [13]. Donors from this group release H_2_S in the presence of thiols such as glutathione (GSH), which are almost exclusively present in biological fluids. They also work in the micromolar range concentration, different from the better characterized and used GYY4137. Our results show that TAGDD have significant antiviral activity against RSV in vitro and they inhibit lung inflammation and reduce viral replication in a mouse model of infection, leading to significant amelioration of clinical illness, therefore they represent a potential novel pharmacological approach to ameliorate RSV-induced lung disease.

## 2. Materials and Methods

### 2.1. Materials

TAGDD-1, -4, and -6 were synthesized and characterized as previously described [13]. TAGDDs 100 mM stocks were prepared in dimethylsulfoxide (DMSO) and diluted in serum free medium at least a thousand fold for the experiments performed in vitro.

### 2.2. Cell Culture

A549 cells, a human alveolar type II-like epithelial cell line, and HEp-2 cells, a laryngeal carcinoma-derived cell line, were obtained from the American Type Culture Collection, Manassas, VA, USA, and were grown in F12K and Minimum Essential Media (MEM) growth medium respectively, containing 10% (*v*/*v*) fetal bovine serum (FBS), 10 mM glutamine, 100 IU/mL penicillin and 100 μg/mL streptomycin.

### 2.3. Virus Preparation and Titration

The RSV Long strain was grown in HEp-2 cells and purified by centrifugation on discontinuous sucrose gradients, as described [14,15], and viral pools were titered in plaque forming units (PFU)/mL using a methylcellulose plaque assay, as described [16]. No contaminating cytokines or lipopolysaccharides (LPS), tested by the limulus hemocyanin agglutination assay, were found in these viral preparations. Virus pools were aliquoted, quick-frozen on dry ice/alcohol and stored at −80 °C until used.

### 2.4. Determination of Lactate Dehydrogenase Activity

Lactate dehydrogenase (LDH) activity in the medium, an index of cellular damage, was measured by colorimetric assay using a commercially available kit (Cayman Chemical, Ann Arbor, MI, USA) following the manufacturer’s instructions. This assay measures cell death in response to chemical compounds or environmental factors using a coupled two-step reaction. In the first step, LDH catalyzes the reduction of nicotinamide adenine dinucleotide (NAD^+^) to NADH and H^+^ by oxidation of lactate to pyruvate. In the second step of the reaction, diaphorase uses the newly-formed NADH and H^+^ to catalyze the reduction of a tetrazolium salt (INT) to highly-colored formazan which absorbs at 490–520 nm. The amount of formazan produced is proportional to the amount of LDH released into the culture medium as a result of cytotoxicity. The results of each experiment are calculated as “% cytotoxicity”, or a percentage of the total amount of LDH contained within the target cells. Thus, for each experiment there is a set of control wells in which all of the target cells are killed using 10% Triton X-100 solution provided in the kit. These are the “maximum release” wells. Also, in each experiment it is necessary to have a set of control wells in which no cytotoxic agents or cytotoxic cells are added, resulting in only the lowest possible (spontaneous or background) LDH release. These are the “spontaneous release” wells. Cells treated with cytotoxic agents or cytotoxic cells will release an amount of LDH that falls between the maximum release level and the spontaneous release level. To calculate % cytotoxicity, LDH activity of the Spontaneous LDH Release Control is subtracted from the chemical-treated sample LDH activity, divided by the total LDH activity ((Maximum LDH Release Control activity)—(Spontaneous LDH Release Control activity), and multiplied by 100.

### 2.5. Quantitative Real-Time PCR

Total RNA was extracted using ToTALLY RNA kit from Ambion (Cat # AM1910, Austin, TX, USA). RNA samples were quantified using a Nanodrop Spectrophotometer (Thermo Fisher Scientific Inc., Wilmington, DE, USA), and quality was analyzed on RNA Nano or Pico chip using the Agilent 2100 Bioanalyzer (Agilent Technologies, Santa Clara, CA, USA). Synthesis of cDNA was performed with 1 µg of total RNA in a 20-µL reaction using the Taqman Reverse Transcription Reagents Kit from ABI (Applied Biosystems, cat. #N8080234, Foster City, CA, USA). The reaction conditions were as follows: 25 °C 10 min, 48 °C 30 min, 95 °C 5 min. Quantitative real-time PCR amplification (performed in triplicate) was done with 1 µL of cDNA in a total volume of 25 µL using the Faststart Universal SYBR Green Master Mix (Roche Applied Science cat. #04913850001, Penzberg, Germany). The final concentration of the primers was 300 nM. 18S RNA was used as housekeeping gene for normalization. PCR assays were run in the ABI Prism 7500 Sequence Detection System (Applied Biosystems, Foster City, CA, USA) with the following conditions: 50 °C 2 min, 95 °C 10 min and then 95 °C 15 s, 60 °C 1 min for 40 cycles.

RSV N-specific RT primer contained a tag sequence from the bacterial chloroamphenicol resistance gene to generate the cDNA, because of self-priming exhibited by RSV RNA. Duplicate cycle threshold (CT) values were analyzed in Microsoft Excel by the comparative CT (ΔΔ*C*T) method as described by the manufacturer (Applied Biosystems). The amount of target (2^−ΔΔ*C*T^) was obtained by normalizing to endogenous reference (18S) sample. The underlined regions in the following sequence indicate the annealing sites of two primers to be used in a polymerase chain reaction. To detect RSV N transcript, we used RSV N dT + Tag (RT primer): CTGCGATGAGTGGCAGGCTTTTTTTTTTTTAACTY-AAAGCTC Cmr Tag. For PCR assay, RSV Tag (R primer): CTGCGATGAGTGGCAGGC. RSV N forward primer: ACTACAGTGT-ATTAGACTTRACAGCAGAAG.

To detect genome (-) strand, we used RSV N + Tag F (RT primer): 5′ CTGCGATGAGTGGCAGGCACTACAGTGTATTAGACTTRA-CAGCAGAAG 3′ Cmr Tag. For PCR assay, RSV Tag: CTGCGATGAGTGGCAGGC. RSV P R#2 primer: GCATCTTCTCCATGRAATTCAGG.

### 2.6. Western Blotting

Total cell lysates were prepared from uninfected and infected A549 cells by adding ice-cold lysis buffer (50 mM Tris-HCl, pH 7.4, 150 mM NaCl, 1 mM EGTA, 0.25% sodium deoxycholate, 1 mM Na_3_VO_4_, 1 mM NaF, 1% Triton X-100 and 1 μg/mL of aprotinin, leupeptin and pepstatin). After incubation on ice for 10 min, the lysates were collected and detergent insoluble materials were removed by centrifugation at 4 °C at 14,000× *g*. Proteins (10 to 20 μg per sample) were then boiled in 2× Laemmli buffer and resolved on SDS-PAGE. Proteins were transferred onto Hybond-polyvinylidene difluoride membrane (Amersham, Piscataway, NJ, USA) and nonspecific binding sites were blocked by immersing the membrane in Tris-buffered saline-Tween (TBST) containing 5% skim milk powder for 30 min. After a short wash in TBST, membranes were incubated with goat anti-RSV polyclonal antibody from Ab D SeroTec for 1 h at room temperature, followed by horseradish peroxidase (HRP)-conjugated secondary antibody (Sigma, St. Louis, MO, USA) diluted 1:10,000 in TBST for 30 min at room temperature. After washing, proteins were detected using an enhanced chemiluminescence system (RPN 2016, GE Healthcare, Amersham, UK) and visualized through autoradiography.

### 2.7. Animal Studies

Ten to 12-week old female BALB/c mice were given H_2_S donor or vehicle (DMSO) 1 h prior to infection and at 6 and 20 h post-infection (p.i.), and infected intranasally (i.n.) with RSV (5 × 10^6^ PFU/mouse). As mock treatment, mice were treated as above and inoculated with an equivalent volume of phosphate buffered saline (PBS). As our model of infection results in pneumonia within the first 24 h, given the large inoculum dose, we did not treat animal past that time. Clinical disease, evaluated by body weight loss and clinical illness score, lung function, lung inflammation and viral replication were used as outcomes [17,18]. Clinical disease will be determined by daily assessment of body weight loss and a clinical severity score on a 0-to-5 grading scale (0 = healthy, 1 = barely ruffled fur, 2 = ruffled fur but active, 3 = ruffled fur and inactive, 4 = ruffled fur, inactive and hunched, 5 = dead). Viral-induced airway dysfunction was measured at baseline and after methacholine challenge using whole-body plethysmography (Buxco, Troy, NY, USA). Virus replication in the lung was measured by plaque assay at peak of replication on day 5 p.i. BAL fluid was collected at day 1 p.i. and used to evaluate total and differential cell counts and to measure cytokines and chemokines using the Bio-Plex Cytokine Mouse Multi-Plex panel (Bio-Rad Laboratories, Hercules, CA, USA). All procedures involving mice were performed in accordance with the recommendations in the Guide for the Care and Use of Laboratory Animals of the National Institutes of Health. The protocol was approved by the Institutional Animal Care and Use Committee of the University of Texas Medical Branch at Galveston (9001002 I, triennial renewal 1 January 2016).

### 2.8. Statistical Analysis

Unpaired parametrical (*t*-test) or non-parametrical (Mann–Whitney) tests were used to compare two groups at a time. One-way analysis of variance (ANOVA) with Tukey’s post-hoc analysis was used in multiple comparisons. Null hypotheses were rejected at *p* values less than 0.05. All data are presented in figures represent means ± standard error (* *p* < 0.05). Experiments were performed a minimum of three times. Statistical analysis was performed with GraphPad Prism 5 software (GraphPad Software, Inc., La Jolla, CA, USA).

## 3. Results

### 3.1. Effect of TAGDDs on RSV Replication In Vitro

**Figure 1:**
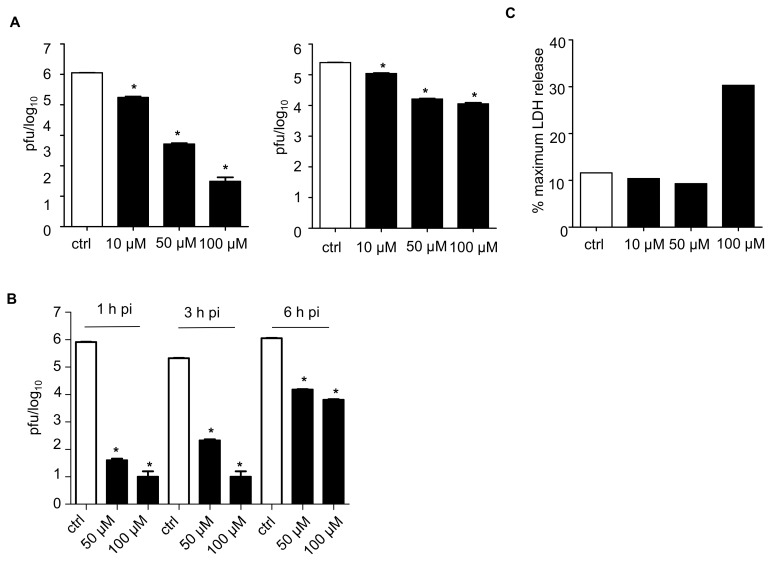
Thiol-activated gem-dithiol H_2_S donors inhibit respiratory syncytial virus (RSV) replication in vitro. (**A**) Confluent monolayers of HEp-2 cells were infected with RSV multiplicity of infection (MOI) 0.01 and treated with 10, 50 or 100 μM of thiol-activated *gem*-dithiol H_2_S donor thiol-activated gem-dithiol-1(TAGDD-1) at 1 h p.i. Cell supernatants (left panel) and cell pellets (right panel) were harvested separately at 24 h p.i to determine viral titer by plaque assay. (**B**) Confluent monolayers of HEp-2 cells were infected with RSV MOI 0.01 and TAGDD-1 was added at 1, 3, and 6 h p.i. at 50 and 100 μM concentrations. The supernatant was collected 24 h later and plaque assay was used to measure RSV titer. The values represent the means of logarithmically-transformed titer values, error bars—standard error of means (SEM), *n* = 3; * *p* < 0.05 compared to control RSV-infected samples using one-way ANOVA with Tukey post-hoc test. (**C**) Cytotoxicity assay of TAGDD-1 in HEp-2 cells using lactate dehydrogenase (LDH) assay.

To investigate the effect of TAGDDs on viral replication, HEp-2 cells were infected with RSV at MOI of 0.01 and treated at 1 h post-infection (p.i.), following removal of inoculum, with TAGDD-1 at 1, 10, 50, or 100 μM concentrations throughout the course of infection. The cell supernatant and cell-associated virus was assessed at 24 h p.i. using plaque assay. There was no significant antiviral effect at the 1 μM concentration, while all the other three concentrations significantly reduced virus titers, with 50 and 100 μM more effective than the 10 μM. Reduction in virus titers was significantly higher for the virus released in the supernatant than for the one that was cell-associated (Figure 1A). A similar result was also obtained when using two other members of the TAGDD family, TAGDD-4 and -6 [13]. There was no effect on viral replication when the donors were given before infection or during adsorption only, but not during infection, indicating that TAGDDs do not affect viral entry.

To investigate whether the antiviral activity was observed if the donor was administered several hours after infection, cells were treated at 3 and 6 h p.i. and harvested to measure viral replication (Figure 1B). TAGDD-1 significantly inhibited the release of RSV into supernatant at all three time points, with earlier administration at 1 and 3 h p.i. resulting in larger reduction of RSV titer (between 3 and 4 logs reduction) than at 6 h (~2 log reduction).

Lactate dehydrogenase (LDH) activity in the medium, an index of cellular damage, was measured by colorimetric assay using a commercially available kit (Cayman Chemical, Ann Arbor, MI, USA) following manufacturer’s instructions. There was no notable cytotoxic effect of TAGDD-1 at concentrations of 10 and 50 μM, while the 100 μM dose induced a 3-fold increase in LDH release in treated cells (Figure 1C). Similar results were observed in Hela cells [13] and in A549 and Vero cells.

**Figure 2:**
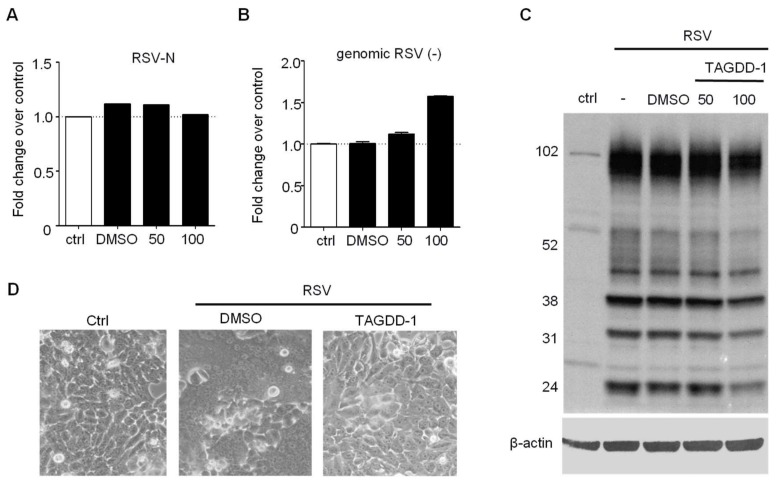
TAGDD-1 does not affect initial steps of RSV replication. A549 cells were infected with RSV MOI 3 and treated with TAGDD-1 at 50 and 100 μM concentrations at 1 h after infection. The cells were harvested 24 h later to extract total RNA (**A**,**B**) or cellular protein (**C**). (**A**) Real-time PCR for RSV-N transcripts normalized to control RSV only using 2^−ΔΔ*C*t^ method (**B**) Absolute quantification by real-time PCR for genomic RSV (-) RNA transcripts normalized to control RSV only. (**C**). Western blot for RSV proteins. Total of 30 μg of protein was used per lane. The membrane was probed using rabbit polyclonal anti-whole RSV antibody (Bio-Rad, Hercules, CA, USA). The membrane was re-probed with anti-human β-actin antibody for loading control. (**D**) Light microscopy photograph (20×) of HEp-2 cells uninfected and infected with RSV MOI of 0.01 for 48 h in the presence of DMSO or TAGDD-1 at 50 μM concentration.

To further investigate the effect of TAGGD treatment on different steps of RSV replication, we assessed viral mRNA, viral genome and protein expression during a single cycle replication, as previously described [8]. A549 cells were infected with RSV at MOI of 1 in the presence or absence of 50 or 100 μM TAGDD-1 and harvested at 24 h p.i. for total RNA and cell protein extraction. There was no decrease in the number of RSV N gene and genome copies (Figure 2A,B), with a mild reduction of some viral protein expression at the 100 μM concentration, possibly due to some cell toxicity (Figure 2C). Similar results were obtained in HEp-2 cells. In addition, we observed a striking reduction in RSV-induced cellular syncytia formation (middle panel vs right panel of Figure 2D), indicating that TAGDD-1 can significantly affect viral-induced cellular fusion.

### 3.2. Effect of TAGDD-1 on RSV Infection In Vivo

**Figure 3:**
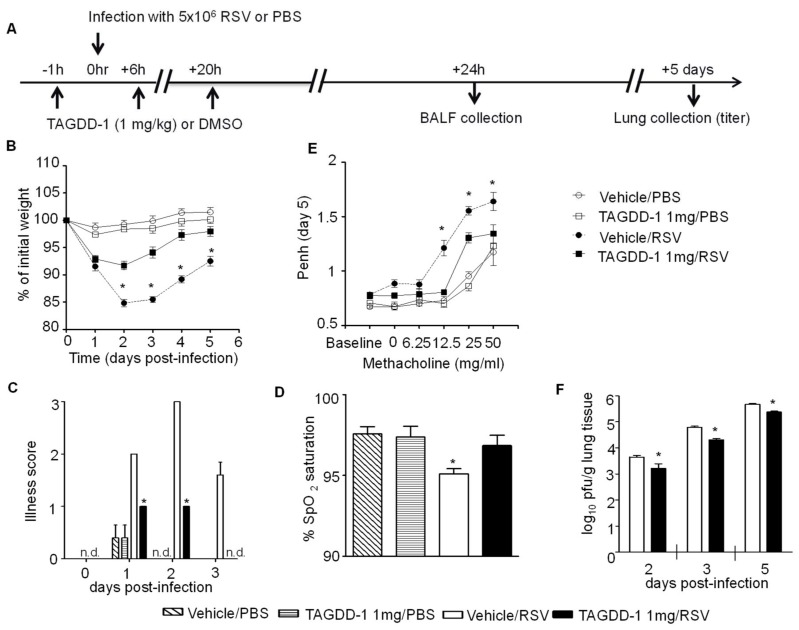
TAGDD-1 treatment attenuates RSV-induced disease and viral titer and ameliorates pulmonary lung function in mice. (**A**) Mice were treated i.n. with different TAGDD-1 (1 mg/kg body weight) or an appropriate volume of vehicle (DMSO) 1 h before, 6 h and 20 h after infection. Mice were inoculated with either RSV or PBS. Mice were monitored daily for changes in body weight (**B**) and disease manifestation (**C**). Body weight (**B**) was calculated based on the original weight before the infection. Black squares—RSV infected TAGDD-1 treated animals; black circles—RSV-infected DMSO (vehicle)-treated animals; open squares—sham PBS-infected TAGDD-1 treated animals; open circles—sham PBS-infected and vehicle (DMSO)-treated animals. Clinical disease score (**C**), calculated on a 0–5 scale where 5 equals death of the animal, was assigned in a blind manner. (**D**) Oxygen saturation levels (SpO_2_) as determined by pulse-oximetry at day 3 after infection. (**E**) Unrestrained, whole-body plethysmography (Buxco Electronics, Inc., Sharon, CT, USA) was used to measure the Enhanced Pause (Penh) to evaluate airway hyperresponsiveness (AHR). Baseline and post-methacholine challenge Penh values were determined at day 5 after infection. (**F**) At day 2, 3 and 5 p.i., lungs were excised and viral replication was determined by plaque assay. Data are expressed as mean ± SEM (*n* = 4–5 mice/group). * *p* < 0.05, n.d.—not determined when TAGDD-1/RSV was compared to vehicle/RSV using one-way ANOVA with Tukey post-hoc test.

To evaluate the potential therapeutic effect of TAGDD-1 treatment in vivo, we used our established mouse model of RSV infection to measure body weight loss, oxygen saturation levels, airway hyperresponsiveness (AHR), virus replication, and lung inflammatory responses [9]. Ten- to 12-week-old female BALB/c mice were given the H_2_S donor (using a 100 mM stock solution) at the dose of either 0.1, 1 or 5 mg/kg or vehicle (DMSO) 1 h prior to infection and at 6 and 20 h p.i., and infected i.n. with RSV (5 × 10^6^ PFU/mouse) (Figure 3A). TAGDD-1 or equal amount of DMSO were diluted in 50 μL PBS for inoculation. The dose range was based on the effective dose of GYY4137 in the same RSV mouse model (50 mg/kg) [9], taking into consideration that the H_2_S release from GYY 4137 in vitro is usually 50 to 100 fold less than TAGDD-1 [11]. As mock treatment, mice were treated as above and inoculated with an equivalent volume of PBS. Mice from all groups were monitored daily for clinical signs of disease and changes in body weight, as previously described [18], while some were harvested to analyze lung tissue and BAL fluid. Uninfected mice treated with either TAGDD-1 or DMSO did not display any signs of significant sickness or weight loss over the 5 days monitoring period. In RSV-infected mice, TAGDD-1 treatment significantly attenuated body weight loss at the dose of 1 mg/kg (Figure 3B), as well as signs of disease (illness score) (Figure 3C), indicating that this treatment is effective in modulating RSV-associated clinical disease. There was no significant effect at the dose of 0.1 mg/kg and the dose of 5 mg/kg did not show any increased benefit, therefore all the subsequent experiments were performed at the dose of 1 mg/kg.

MouseOx™ Pulse-oximeter (STARR Life Sciences, PA, USA) and AHR in response to methacholine challenge by whole-body plethysmography (Buxco Electronics, Inc., Sharon, CT, USA) were used to determine the effect of TAGDD-1 treatment on pulmonary lung function. Pulse oximetry is widely used in people with acute and chronic respiratory conditions to provide an important clinical readout of morbidity in respiratory diseases [19]. Recent advances in probe design and software analysis now make pulse oximetry feasible as a noninvasive assessment of lung damage in monitoring murine models of viral infections [20,21]. SpO_2_ measurement was performed on day 3 p.i., based on optimization studies of pulse oximetry in our mouse model of RSV infection, during which SpO_2_ levels in infected mice were significantly decreased and different from control mock mice. We observed no significant difference between mock vehicle and TAGDD-1 treated mice which maintained >97% SpO_2_ levels. However, RSV vehicle-infected mice exhibited significantly marked decreases in SpO_2_ levels (<95%), compared with TAGDD-1-treated infected mice (Figure 3D).

Next, we measured AHR in response to methacholine challenge in unrestrained mice using a whole body plethysmograph, as previously described [18]. At day 5 p.i. mice from were exposed for 2 min to aerosolized saline and subsequently to increasing concentrations of aerosolized methacholine (0, 6.25, 12.5, 25, and 50 mg/mL in isotonic saline, Sigma, St. Louis, MO, USA). Following each nebulization, Penh were recorded for 3 min, averaged, and expressed for each dose of methacholine. TAGDD-1 treatment did not alter baseline Penh values or AHR to methacholine in mock-infected animals. No differences in AHR were observed between the TAGDD-1 and vehicle RSV infected groups at the lowest methacholine dose of 6.25 mg/mL, while TAGDD-1 treatment strongly attenuated RSV-induced AHR at all the higher doses of methacholine tested (Figure 3E).

Finally, we examined virus replication at day 2, 3, and 5 p.i., the latter representing the peak viral titer [18], measured by plaque assay of lung homogenates. TAGDD-1 treated mice showed an average of half log decrease in virus lung titer at all time points tested, compared to the vehicle treated-infected animals (Figure 3F).

**Figure 4:**
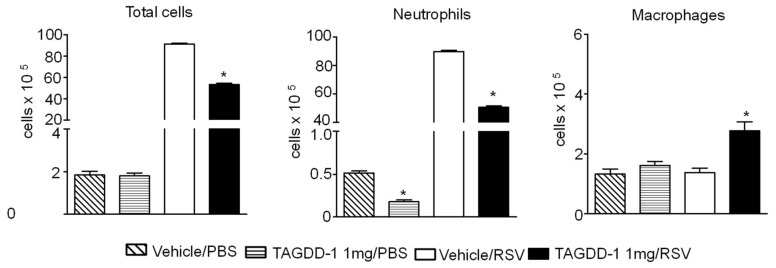
TAGDD-1 reduces airway cellular infiltration after RSV infection. Mice were treated i.n. with TAGDD-1 (1 mg/kg body weight) or an appropriate volume of vehicle (DMSO) 1 h before, 6 h and 20 h after infection with RSV. Bronchoalveolar lavage fluid (BAL) was collected at 1 day p.i. to determine differential cell counts (neutrophils and macrophages) by analysis of Protocol HEMA3 stained cytospins. Data are expressed as mean ± SEM (*n* = 4–5 mice/group). * *p* < 0.05, compared to vehicle/PBS or vehicle/RSV group using one-way ANOVA with Tukey post-hoc test.

**Figure 5:**
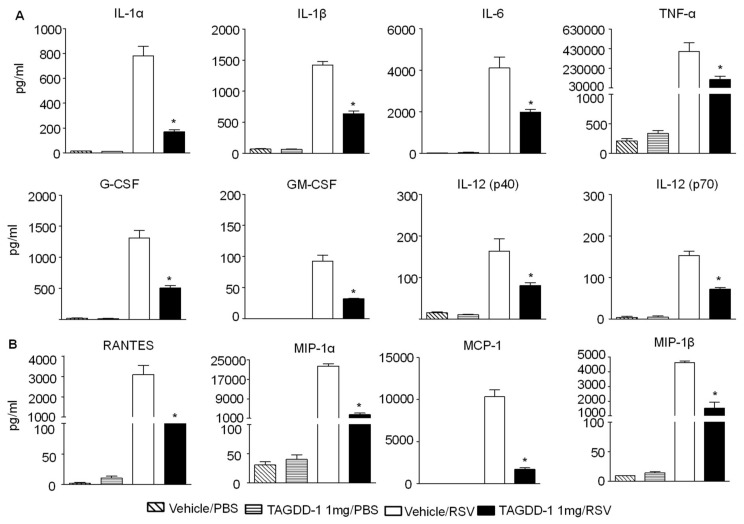
TAGDD-1 inhibits secretion of cytokines and chemokines in response to RSV infection. Mice were treated with TAGDD-1 or vehicle (DMSO), and infected with RSV or sham-infected with PBS. The bronchoalveolar lavage was harvested at day 1 p.i. to measure (**A**) cytokines and (**B**) chemokines by multi-Plex Cytokine detection system. The bar graph represents mean ± SEM (*n* = 4–5 mice/group). * *p* < 0.05, compared to vehicle/RSV group.

To determine whether TAGDD-1 administration could modulate RSV-induced lung inflammation, animals treated with TAGDD-1 1 h before, 6 h and 20 h p.i. or vehicle and mock- or RSV-infected were sacrificed at day 1 p.i. to collect BAL samples for total and differential cell count and for measurement of cytokine and chemokines. We observed a significant reduction of cellular infiltration (~41.5%) at day 1 p.i. by TAGDD-1 treatment in RSV-infected mice, compared with vehicle alone (Figure 4), which was accounted mostly by a reduction in neutrophils recruited to the airways (vehicle (89.87 × 10^5^ ± 0.78) vs. TAGDD-1 (50.58 × 10^5^ ± 1.04) RSV-infected mice), with a concomitant increase in the macrophage population. In addition, TAGDD-1 treatment significantly decreased the production of the proinflammatory cytokines IL-1α, IL-1β, IL-6, TNF-α, granulocyte-macrophage colony-stimulating factor (GM-CSF) and granulocyte-colony stimulating factor (G-CSF), as well as (Figure 5A), as well as the release of the chemokines RANTES, MIP-1α, MIP-1β, MCP-1, and KC (Figure 5B) in RSV-infected mice.

## 4. Discussion

RSV is a primary cause of severe lower respiratory tract infections in children, as well as in other populations, leading to increased morbidity and mortality. Currently, there is no available vaccine or treatment for RSV infection, beside supportive measures. The viral-induced lung inflammatory response, triggered by secretion of cytokine and chemokine from viral-infected airway resident cells, such as airway epithelial cells and alveolar macrophages, plays an important role in disease pathogenesis. We and others have shown that modulation of the inflammatory response is associated with amelioration of clinical illness in animal models of RSV infection [18,22,23,24], making it an important target for the development of effective treatment strategies. Recent studies have also pointed to an important correlation between viral replication and RSV disease outcome, as infants with greater viral quantities in the respiratory tract secretions have been shown to be at greater risk for prolonged hospitalization, intensive care unit stay and mechanical ventilation [25,26], and infants with less rapid clearance of RSV have greater disease severity, therefore approaches that combine inhibition of proinflammatory responses with the inhibition of viral replication would be likely the most effective in modulating severe lung disease associated with RSV infection.

H_2_S is an endogenous gaseous mediator that regulates many intracellular signaling pathways, playing a particular important role in cytoprotective, anti-inflammatory, and antioxidant cellular responses (reviewed in [27]). Decreased H_2_S lung and serum levels have been associated with the pathophysiology of acute and chronic lung diseases such asthma and chronic obstructive pulmonary disease [1]. We have recently shown for the first time that levels of intracellular H_2_S modulates cellular inflammatory responses and viral replication in in vitro and in vivo models of RSV infection [8,9]. A number of H_2_S donors exist which include inorganic salts, compounds with organic backbones, amino-acids and naturally occurring compounds (reviewed in [7]). Inorganic salts, such as sodium hydrosulfide (NaHS), are the H_2_S donors most commonly used to investigate the therapeutics effects of H_2_S administration in vitro and in vivo and have been tested in a variety of respiratory diseases. They are the most cost-effective but least controllable H_2_S donors available, due to an immediate release of H_2_S in solution or biological fluid, creating challenges due to potentially toxic effects on the studied system. GYY4137, a Lawesson’s reagent derivative, is a water-soluble H_2_S donor which shows a slow (in the micromolar range when used at millimolar concentrations) release of H_2_S by hydrolysis once in solution in a pH-dependent manner [11]. This compound has been widely used and it has shown anti-inflammatory properties in cultured cells and in a variety of animal models of inflammation in vivo [28]. We recently showed that administration of GYY4137 strongly inhibits replication of paramyxoviruses, as well as, other RNA enveloped viruses [8,12] and results in improvement of lung function and disease outcome in a mouse model of RSV infection [9].

In the past few years, there has been an increased effort in synthesizing H_2_S donors that release H_2_S in the presence of thiols, such as TAGDDs, because, unlike hydrolysis-based H_2_S donors (i.e., GYY4137 and Na_2_S/NaHS), TAGDDs are stable in aqueous solutions and they do not release H_2_S upon hydrolysis. However, they do show a time-dependent H_2_S generation in the presence of cysteine and GSH, which are found only in cells and body fluids. This quality allows for better controlled release of H_2_S, which will not be affected by the preparation of the compound prior to the administration. Our results show that TAGDD-1 treatment had no effect on the initial steps of virus replication, as there was no reduction in RSV genome replication, viral mRNA and protein synthesis. It highly reduced the amount of infectious virus present in the cell supernatant, with a much less robust effect on cell-associated virus, suggesting that TAGDD-1 treatment inhibits viral replication in part at the level of virus assembly, but mostly at the level of virus release. TAGDD-1 treatment also dramatically reduced RSV-induced syncytia formation, suggesting that TAGDD-1 can affect F protein structure and/or cellular localization. An important finding was that the concentration of the most effective antiviral dose in vitro was 100 fold lower (50 μM) than the one required by GYY4137 for a similar effect, which was between 5 to 10 mM [8,12]. TAGDD-1 intranasal delivery to RSV-infected mice markedly improved clinical disease parameters and pulmonary dysfunction compared to vehicle treated controls, although it was less effective in reducing viral replication. It is important to point out that in small animal models modulation of RSV-induced lung inflammation, although beneficial in ameliorating disease outcomes, is associated with increase in virus replication [18,29], which would be of clinical relevance in the human population. Therefore, the finding that TAGDD-1 can reduce virus titers while inhibiting inflammatory responses is quite significant. Further research is required to better understand the discrepancy of the antiviral effect observed in vitro conditions vs in vivo treatment. One possibility is the fact that RSV induces GSH depletion in the course of infection [30], which would limit the release of H_2_S from TAGGD-1 after administration.

## 5. Conclusions

Here, we report that treatment with TAGDD-1 exerts potent antiviral activity in vitro and markedly protected against RSV-induced illness and lung inflammation. These results support our previous findings that administration of exogenous H_2_S donors can play a critical role in modulating RSV-induced disease and should be considered as a potential novel target for treatment of lung disease.

## 6. Patents

9,504,701 of 3 March 2016 (Casola A., Escaffree, O., Freiberg, A., Garofalo R.P.) Methods for Treating Viral Infections Using Hydrogen Sulfide Donors.
